# Chronically endurance‐trained individuals preserve skeletal muscle mitochondrial gene expression with age but differences within age groups remain

**DOI:** 10.14814/phy2.12239

**Published:** 2014-12-18

**Authors:** Matthew L. Johnson, Ian R. Lanza, Daniel K. Short, Yan W. Asmann, K. Sreekumaran Nair

**Affiliations:** 1 Division of Endocrinology and Metabolism, Mayo Clinic College of Medicine, 200 First St SW, Rochester, 55905, Minnesota

**Keywords:** Aging, exercise, mitochondria, oxidative damage, proteasome, sarcopenia

## Abstract

Maintenance of musculoskeletal function in older adults is critically important for preserving cardiorespiratory function and health span. Aerobic endurance training (ET) improves skeletal muscle metabolic function including age‐related declines in muscle mitochondrial function. To further understand the underlying mechanism of enhanced muscle function with ET, we profiled the gene transcription (mRNA levels) patterns by gene array and determined the canonical pathways associated with skeletal muscle aging in a cross‐sectional study involving vastus lateralis muscle biopsy samples of four subgroups (young and old, trained, and untrained). We first analyzed the sedentary individuals and then sought to identify the pathways impacted by long‐term ET (>4 years) and determined the age effect. We found that skeletal muscle aging in older sedentary adults decreased mitochondrial genes and pathways involved in oxidative phosphorylation while elevating pathways in redox homeostasis. In older adults compared to their younger counterparts who chronically perform ET however, those differences were absent. ET did, however, impact nearly twice as many genes in younger compared to older participants including downregulation of gene transcripts involved in protein ubiquitination and the ERK/MAPK pathways. This study demonstrates that in individuals who are chronically endurance trained, the transcriptional profile is normalized for mitochondrial genes but aging impacts the number of genes that respond to ET including many involved in protein homeostasis and cellular stress.

## Abstract (precis)

e12239

Transcriptional patterns in skeletal muscle are distinct in older compared to young adults. Although chronic endurance training in older adults restores the majority of the mitochondria transcripts to expression levels similar to young, there are many genes involved in protein homeostasis and cellular stress that exhibit attenuated responses to exercise in old compared to young adults.

## Introduction

Currently, an estimated 39 million Americans are age 65 and older, an increase of 50% over the last 30 years and now the fastest growing segment of the population (Manton and Vaupel 1995; Bureau USC 2014). Low levels of physical activity with age not only increase dependence but predispose older adults to cardiovascular, metabolic, and other chronic diseases (Paffenbarger et al. 1986; Lee et al. 1995). Maintenance of musculoskeletal function in older adults is, therefore, critically important for preserving health span. Aerobic endurance training (ET) increases skeletal muscle function in both young and older adults (Short et al. 2003), improves well‐being and reduces cardiometabolic risk, thereby acting as a countermeasure to many age‐associated processes (Paffenbarger et al. 1986; Lee et al. 1995).

The maximal rate at which an individual consumes oxygen (VO_2_max), the major metric of whole‐body cardiorespiratory fitness, declines with age even after correcting for losses in lean mass (Rogers et al. 1990; Short et al. 2005). While aging adversely affects central hemodynamics, a key determinant of VO_2_max, sedentary older adults also have a lower arterio‐venous difference in the extraction of oxygen across active muscles at VO_2_max compared to their younger counterparts (Julius et al. 1967). Those data implicate deterioration of skeletal muscle oxidative capacity as an important component of reduced VO_2_max with age. In vivo measurements indicate a decline in both maximal (MAPR) and resting ATP production rate (Conley et al. 2000; Petersen et al. 2003). Ex vivo (biopsy) measurements support those data showing a decline in rates of mitochondrial protein synthesis, mitochondrial content, and activities of key tricarboxylic acid cycle (TCA) enzymes with age in sedentary adults (Rogers et al. 1990; Rooyackers et al. 1996; Tonkonogi et al. 2003; Short et al. 2005; Lanza et al. 2008). Short‐term ET, however, can reverse the decline in TCA enzyme activities and protein synthesis (Short et al. 2003).

We previously compared young and older men and women (*N* = 42) who were sedentary to those who are chronically endurance trained (ET) at a high level and found that ET maintained insulin sensitivity independent of age (Lanza et al. 2008). We also reported age‐related declines in markers of mitochondrial function in the sedentary groups, and while chronic ET prevented the decline in MAPR, age effects persisted for several markers including mtDNA abundance, the expression of nuclear transcription factors and several mitochondrial proteins. The mechanisms underlying this persistent age effect on mitochondria in spite of maintained high physical activity levels are unclear. To this end, transcriptional profiling is a useful approach to elucidating potential mechanisms to explain this phenomenon.

In the present study, we measured the gene transcript profile (mRNA levels) of this well characterized cohort. Several previous studies have assessed gene expression in human skeletal muscle in the context of aging (Welle et al. 2004; Zahn et al. 2006; Melov et al. 2007; Raue et al. 2012; Phillips et al. 2013). These studies reported that aging affects posttranscriptional processes (Phillips et al. 2013), increases the expression of genes related to inflammation (Giresi et al. 2005), oxidative stress (Welle et al. 2004), and protein degradation (Welle et al. 2003) while decreasing genes related to mitochondrial function (Welle et al. 2003; Zahn et al. 2006; Melov et al. 2007). To date, two resistance training studies have investigated the interaction of age and exercise (Melov et al. 2007; Phillips et al. 2013) and suggest that mitochondrial gene expression can be normalized with an exercise‐training program independent of age. However, in the context of aging, the effect of chronic ET (>4 years) on gene expression patterns has not been characterized.

The objective of the current study was to first characterize the gene expression patterns and canonical pathways associated with skeletal muscle aging in sedentary individuals, then to identify the pathways affected by long‐term ET and if the transcriptional response to exercise differed by age.

## Materials and Methods

### Study population

Forty men and women were recruited from the local Olmsted County community. Twenty were young (18–30 years) participants and further divided into young sedentary (YS, *N* = 4 women, six men) and young trained (YT, *N* = 4 women, six men). Twenty were older (59–76 years) participants and divided into older sedentary (OS, *N* = 4 women, 6 men) and older trained (OT, *N* = 4 women, 6 men). Sedentary participants engaged in structured physical activity <30 min per day twice per week, while trained participants performed at least 1 h of cycling or running 6 days per week over the past 4 years (confirmed by Leisure‐Time Activity [LTA] questionnaire). An initial screen of medical history, physical exam, graded exercise treadmill test, and comprehensive blood test was performed to exclude a history of metabolic or cardiovascular disease, plasma glucose >99 mg/dL, body mass index (BMI) >28 kg/m^2^, medications known to affect the outcome measures, anemia, pregnancy, tobacco, alcohol, or other substance abuse. Written informed consent was obtained from all subjects, as approved by the Mayo Foundation Institutional Review Board, in conformance with the standards set by the Declaration of Helsinki.

### Study protocol

The details of this study have been published previously (Lanza et al. 2008). Briefly, after enrollment, two outpatient visits to the Mayo Clinic Clinical Research Unit (CRU) were performed. The first visit included dual X‐ray absorptiometry (Lunar DPX‐L, Lunar Radiation, Madison, WI) to measure total and regional fat and fat‐free mass. Abdominal and visceral fat were measured with computed tomography (Imatron C‐150, San Francisco, CA), as described previously (Giresi et al. 2005). Maximal muscle strength during leg press, arm curl, and chest press was measured as the maximum weight each subject could lift in a single repetition for each muscle group. The participants returned to the CRU for the second outpatient visit for measurement of VO_2_ peak from expired gas analysis during a graded test on a bicycle ergometer. The results of these measurements have been published previously (Lanza et al. 2008).

At least 7 days after the second outpatient testing, participants were provided a weight maintenance diet (55% carbohydrates, 15% protein, and 30% fat) for three consecutive days prior to inpatient testing by the Mayo Clinic CRU. Additionally, participants were instructed to refrain from exercise during this 3‐day period. At 17:00 h on day 3 of the diet, participants were admitted to the CRU for 48 h. At 06:30 the following morning, baseline blood samples and a muscle biopsy (350–400 mg) were obtained from the vastus lateralis muscle under local anesthesia (Giresi et al. 2005). Additional results from these measurements were reported previously (Lanza et al. 2008). In addition, due to the large number of inquires for additional data, Table S1 provides participant data for select outcome measures and supplementary methods are also provided for those outcome measures (see Appendix S1).

### Microarray analysis and statistical analysis

A Qiagen RNeasy Fibrous Tissue Kit (Qiagen, Valencia, CA) was used to extract total RNA from biopsy tissue samples taken from the vastus lateralis. Following treatment with DNase, gene transcript profiles were examined in young and old sedentary and endurance‐trained participants through microarray experiments, as described previously (Welle et al. 2003). Gene transcript profiles were measured by high‐density oligonucleotide microarrays containing probes for 54,675 transcripts and expressed sequence tags (HG‐U133 plus 2.0 GeneChip Arrays, Affymetrix, Santa Clara, CA). Genes that were differentially expressed between young and older sedentary and trained groups were identified as those with *P* values ≤0.01. The arrays were normalized using invariant probe set normalization, and the expression measurement for each transcript was calculated using PM‐only model‐based expression index by dChip (Wing 2013). Genes with all “absent” calls by dChip across all compared samples were removed from further analysis. After dChip's quantile normalization, and PM‐only model‐based expressed summarization, three chips did not pass the QC steps: YT2, YS1, and OT1. These three chips were excluded from further analysis. In addition, we did not consider the genes with average intensities ≤50 in all compared groups. Genes with a *P* value ≤0.01 were considered as potential candidates of differentially expressed genes between the compared groups, and used as the “focus genes” for Ingenuity Pathway Analysis. In order to avoid inflating pathways, only the nonredundant probe sets were used in the focus and reference gene lists. The data set has been deposited in the Gene Expression Omnibus (Accession number, GEO9103; available at www.ncbi.nlm.nih.gov/geo/).

## Results

**Figure 1.**
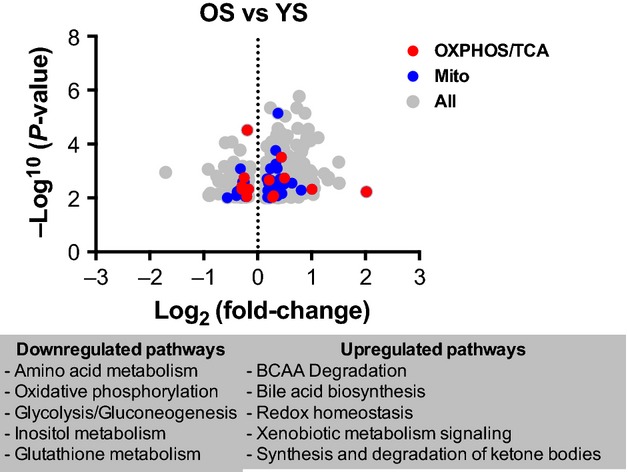
Comparison of skeletal muscle gene expression in young and older participants. A volcano plot of the 625 genes differentially expressed (≤0.01) in skeletal muscle from old sedentary (OS) compared to young sedentary (YS). Genes involved in oxidative phosphorylation/TCA cycle are in red, while those identified as mitochondrial are in blue. Remaining genes are highlighted in grey. The canonical pathways that are associated with significantly (≤0.05) up‐ and downregulated genes are listed using Ingenuity Pathway Analysis (IPA). See also Table S2.

We first compared 10 older sedentary (OS) to 10 younger sedentary (YS) participants to observe the effect of aging on skeletal muscle gene expression in sedentary individuals (Fig. 1). This analysis revealed 625 differentially expressed genes including genes involved in oxidative phosphorylation (OXPHOS) and the tricarboxylic acid cycle (TCA cycle) and mitochondrial function (Table S2). These 625 genes were used as “focus genes” for Ingenuity Pathway Analysis (IPA), which revealed several significantly altered canonical pathways associated with up‐ or downregulated genes in OS compared to YS (Table S3). Particularly downregulation of pathways involved in oxidative and amino acid metabolism and upregulation of pathways involved in BCAA degradation and oxidative stress.

**Figure 2.**
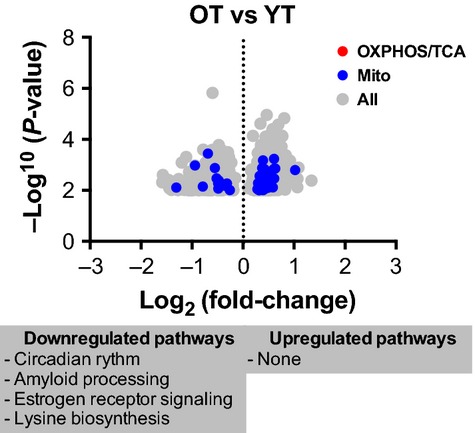
Comparison of skeletal muscle gene expression in older and young chronically ET adults. A volcano plot 1287 genes differentially expressed (≤0.01) in skeletal muscle from old trained (OT) compared to young trained (YT). Genes involved in oxidative phosphorylation/TCA cycle are in red, while those identified as mitochondrial are in blue. Remaining genes are highlighted in grey. The canonical pathways that are associated with significantly (≤0.05) up‐ and downregulated genes are listed using Ingenuity Pathway Analysis (IPA). See also Table S3.

A comparison of OT and YT subjects revealed 1287 differentially expressed genes, although none of the canonical pathways revealed by IPA involved cellular energetics (Fig. 2, Tables S4 and S5).

**Figure 3.**
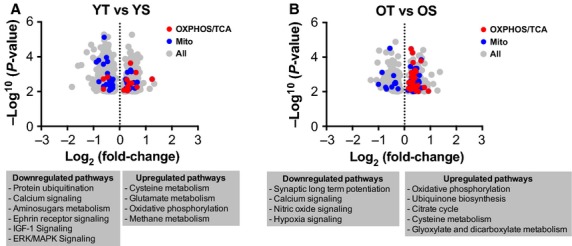
Comparison of skeletal muscle gene expression between sedentary and Chronically ET young and older adults. (A) A volcano plot 1157 genes differentially expressed (≤0.01) in skeletal muscle from young trained (YT) compared to young sedentary (YS) along with canonical pathways that were significantly (≤0.05) up‐ and downregulated. (B) A volcano plot 525 genes differentially expressed (≤0.01) in skeletal muscle from old trained (OT) compared to old sedentary (OS) along with canonical pathways that were significantly (≤0.05) up‐ and downregulated. Genes involved in oxidative phosphorylation/TCA cycle are in red, while those identified as mitochondrial are in blue. Remaining genes are highlighted in grey. Canonical pathways listed using Ingenuity Pathway Analysis (IPA). See also Table S4.

Next we compared the trained and untrained participants’ skeletal muscle gene expression. Comparing ten young trained (YT) to the previous mentioned YS participants and shown in Figure 3A, there were 1157 differentially expressed genes in YT compared to YS subjects (Table S6), with metabolic pathways among the top canonical pathways that were upregulated in young endurance‐trained participants, while protein ubiquitination and the stress response pathway ERK/MAPK signaling were significantly downregulated (Table S7). The number of genes regulated by training was lower in older compared to younger participants with 525 differentially expressed genes reaching the significance threshold of *P* ≤ 0.01 when 10 old trained (OT) were compared to OS subjects (Fig. 3B and Table S8). In older participants training upregulated multiple pathways involved in mitochondrial and oxidative metabolism (Table S9).

**Figure 4.**
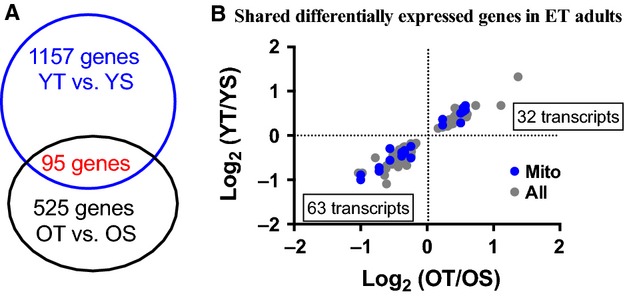
Genes differentially expressed between both young and older ET adults compared to their sedentary counterparts. (A) A Venn diagram showing shared (95) genes altered by training in young and older participants as outlined in Figure 2. (B) A volcano plot of the 95 genes commonly affected by ET between young and older participants. All shared transcripts were changed in a similar fashion by training in young versus older groups (no data points were observed in up‐left and lower right corners).

Figure 4A demonstrates that twice as many genes were different between trained and untrained young participants as compared to older participants (1157 vs. 525) and that training altered 95 genes in common in young and old. Of these 95 common genes, 32 were upregulated and 63 were downregulated with ET in both age groups (Fig. 4B). Eleven of the 95 genes were involved in mitochondrial function.

**Figure 5.**
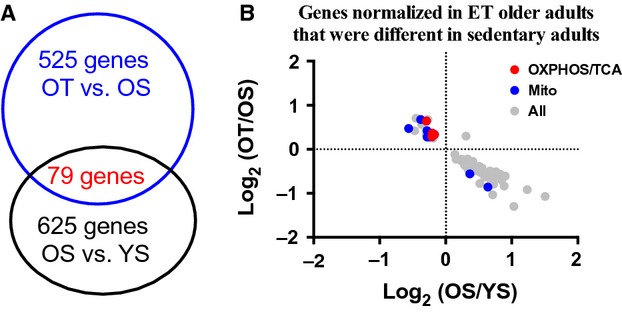
Genes normalized in ET older adults that were differentially expressed in sedentary old adults. (A) A Venn diagram showing genes altered by training in older participants (79) that were initially different between OS and YS. (B) Genes involved in oxidative phosphorylation/TCA cycle are in red, while those identified as mitochondrial are in blue. Remaining genes are highlighted in grey. See also Table S6.

Figure 5A shows that 79 genes were different in trained older participants that were initially different between OS and YS participants. Figure 5B shows that in trained participants all but one of the 79 genes was normalized.

## Discussion

This current study examined the effects that aging and long‐term training status have on skeletal muscle transcription in participants between the ages of 18 and 76 years. When older sedentary adults were compared to their younger counterparts, 625 genes were differentially expressed with subsequent pathway analysis identifying downregulation of oxidative metabolism, glycolysis, and amino acid metabolism and upregulation of BCAA degradation and redox homeostasis pathways. While those differences were absent when a comparison of young and older adults who chronically performed ET was made. Comparing the gene expression patterns of untrained to trained participants revealed that ET altered nearly twice as many genes in young compared to older participants including downregulation of genes involved in protein ubiquination and ERK/MAPK signaling. Independent of age, however, ET increased transcript levels of genes involved in oxidative metabolism and antioxidant defense.

The current study demonstrated that the expression of genes related to mitochondrial function and energy metabolism was expressed at lower levels in OS compared to YS participants. Numerous studies have demonstrated that aging is associated with a reduction in mitochondrial function (Trounce et al. 1989; Boffoli et al. 1994; Proctor et al. 1995; Rooyackers et al. 1996; Houmard et al. 1998; Conley et al. 2000; Petersen et al. 2003; Short et al. 2005), and decreased expression of select genes encoded by both nuclear and mitochondrial DNA (Welle et al. 2004; Zahn et al. 2006; Melov et al. 2007), but increased with exercise independent of age (Melov et al. 2007; Phillips et al. 2013). The global gene expression analysis of the current study supports a notion that ET affects mitochondrial function at the transcriptional level involving multiple genes. The current data also demonstrated an age‐associated increase in antioxidant gene expression related to redox homeostasis and xenobiotic metabolism; specifically catalase and peroxiredoxin 1 and 6 were elevated in OS compared to YS, as were the aldehyde dehydrogenase enzymes (ALDH9A1, ALDH3A2, ALDH6A1, ALDH1L1), which are involved in cellular xenobiotic metabolism. Increased endogenous antioxidant capacity and xenobiotic metabolism with age is noted in several models of aging and associated with increased production of reactive oxygen species in skeletal muscle (McElwee et al. 2004; Gems and McElwee 2005; Lanza et al. 2012). Together these data indicate that sedentary aging produces a gene transcription profile consistent with mitochondrial dysfunction, elevated oxidative stress, and impaired cellular homeostasis.

Genes involved in mitochondrial oxidative phosphorylation were higher in ET participants independent of age a finding consistent with the functional measurements of MAPR in these participants previously reported (Lanza et al. 2008), and the two other reports on gene expression with age and shorter term resistance exercise training (Melov et al. 2007; Phillips et al. 2013). Striking is the remarkable similarity in mitochondrial gene expression and canonical pathways between OT and YT participants. The pathways that remained downregulated included the circadian rhythm pathway known to be expressed in skeletal muscle (Panda et al. 2002; Storch et al. 2002) that when disrupted, such as in BMAL1 deficient mice, result in a progeroid phenotype (Kondratov et al. 2006). Estrogen receptor signaling was also found to be downregulated in OT compared to YT, which is likely related to the postmenopausal status of our older female participants. While the ET participants had similar weekly training volume, VO_2_peak was lower in the OT group (Table S1), therefore, the pathways that remained different between OT and YT participants could be due to aging or differences in training intensity. To better understand the similarities, we compared differences by training status (Fig. 4A and B). Only 95 genes (representative of 18.1% for older subjects, and 8.2% for young subjects) were found to be differentially expressed and shared between trained and untrained participants and while the effects of training (direction of changes) in both age groups are the same for those 95 genes, none are involved in oxidative metabolism, the TCA cycle, or the electron transport chain.

The increased expression of genes related to BCAA degradation in OS participants potentially contributes to the lower fat‐free mass in comparison with the OT previously reported on this cohort (Lanza et al. 2008). A differential expression of genes between trained and untrained groups was observed involving pathways regulating cellular stress response and protein degradation. Specifically, the ubiquitin pathway was robustly downregulated in YT compared to YS, suggesting that the signal for degrading proteins via the ubiquitin proteasome pathway, which degrades damaged proteins (Bota et al. 2002; Pickering and Davies 2012), was lower in YT compared to YS participants. While not all genes identified by the pathway analysis currently have a well‐defined role in skeletal muscle (i.e., BIRC4 or USP11) many including USP19, USP2, and UBR2 are associated with skeletal muscle dysfunction (Wing 2013; Hockerman et al. 2014). Together with a decrease in the MAPK/ERK signaling pathway, which responds to cellular stress (Kim and Choi 1802), is consistent with a decreased need for the clearance of damaged proteins in young ET individuals. Neither of these pathways was significantly affected in older participants (up or down). These data are consistent with the notion that ET may lower the oxidative burden in young but not older participants; a possibility that requires more in‐depth interrogation (Johnson et al. 2014).

Due to the long period of aging after the genetic potential for growth is expressed, examining the interaction between age and exercise on gene expression patterns in the same humans is logistically not practical. However, by comparing the specific genes that were differentially expressed between young and older sedentary groups compared to the OT group offers new insights of interaction between ET and age. In the OT group, this represented 12.6% of genes that are differentially expressed and training normalized 79 of them (Fig. 5). Among these genes increased expression of mitochondrial genes and decreased expression of oxidative stress and DNA damage genes are of substantial interest. These data indicate that ET impacts approximately 10% of genes differentially expressed with age in sedentary individuals and produce a transcript profile more consistent with their younger counterparts (Fig. 5).

In conclusion, skeletal muscle aging in older sedentary adults demonstrated a gene expression pattern consistent with reductions in oxidative metabolism and elevated levels of oxidative stress. These differences were not found however, between trained older and young adults. In addition while those who performed chronic ET did demonstrate increased transcript levels of genes involved in oxidative metabolism in both young and older people, trained young participants compared to their sedentary counterparts exhibited nearly twice as many differentially expressed genes in comparison to older participants including downregulation of gene transcripts involved in protein ubiquitination and the ERK/MAPK pathways.

## Acknowledgments

The authors are greatly indebted to the skillful assistance of Maureen Bigelow, Jane Kahl, Roberta Soderberg, Beth Will, Deborah Sheldon, Kate Klaus, Jill Schimke, Daniel Jakaitis, Dawn Morse, and Melissa Aakre.

## Conflict of Interest

None declared.
